# Reconstituting Immune Surveillance in Breast Cancer: Molecular Pathophysiology and Current Immunotherapy Strategies

**DOI:** 10.3390/ijms222112015

**Published:** 2021-11-06

**Authors:** Chiara Cilibrasi, Panagiotis Papanastasopoulos, Mark Samuels, Georgios Giamas

**Affiliations:** Department of Biochemistry and Biomedicine, School of Life Sciences, University of Sussex, Falmer, Brighton BN1 9QG, UK; ppapanastasopoulos@nhs.net (P.P.); m.samuels@sussex.ac.uk (M.S.)

**Keywords:** breast cancer, immunoediting, immune tumour microenvironment, immunotherapy

## Abstract

Over the past 50 years, breast cancer immunotherapy has emerged as an active field of research, generating novel, targeted treatments for the disease. Immunotherapies carry enormous potential to improve survival in breast cancer, particularly for the subtypes carrying the poorest prognoses. Here, we review the mechanisms by which cancer evades immune destruction as well as the history of breast cancer immunotherapies and recent developments, including clinical trials that have shaped the treatment of the disease with a focus on cell therapies, vaccines, checkpoint inhibitors, and oncolytic viruses.

## 1. Introduction

Breast cancer (BC) is the most commonly diagnosed cancer in women worldwide [1]. Although a great amount of research has focused on improving the diagnosis of the disease and the clinical management of patients, BC is still the leading cause of cancer death in women. Between 20% and 30% of women with BC go on to develop a deadly metastatic disease [2,3].

**Figure 1:**
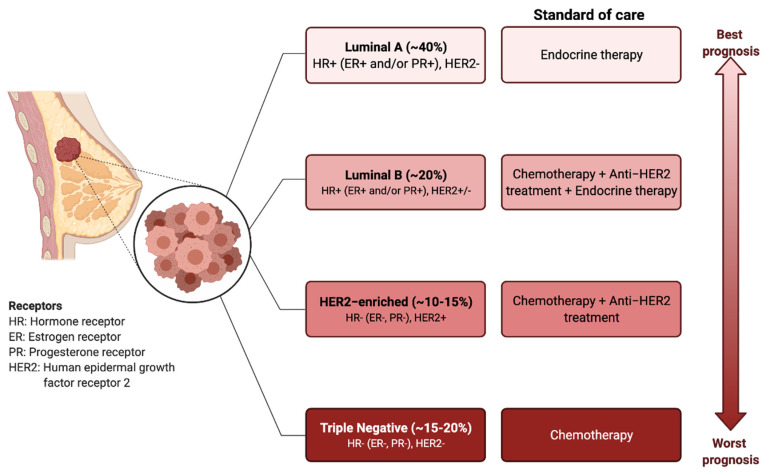
Breast cancer subtypes: prognosis and standard treatments. BC can be classified into four subtypes based on the expression of estrogen receptor (ER), progesterone receptor (PR), and human epidermal growth factor receptor 2 (HER2). Targeted and endocrine therapies are administered based on the molecular markers. The triple-negative breast cancer types (i.e., ER^−^, PR^−^, HER2^−^), have the worst prognosis and do not respond to the endocrine therapies or HER2 targeting agents. Chemotherapy is the only therapeutic regimen used.

BC has traditionally been classified into four different molecular subtypes based on the expression of the estrogen receptor (ER), progesterone receptor (PR), and epidermal growth factor receptor 2 (HER2) [4,5]. The molecular profile together with the histological features of the tumour determine the treatment options, which involve surgical resection, radiotherapy, endocrine therapy, targeted therapies, or systemic chemotherapy [6] (Figure 1).

Over the last few decades, it has become clearer that BC consists not only of a heterogeneous population of neoplastic cells, but also of a variety of resident and infiltrating host cells, secreted factors, extracellular matrix proteins, and extracellular vesicles, collectively known as the tumour microenvironment (TME) [7]. Cross-talk between cancer cells and other cell types functionally sculpts the microenvironment, impacting breast tumour progression and therapeutic effectiveness [8]. The immune system represents an important component of the TME and, alongside the traditional hallmarks of cancer, such as unregulated cell growth and evasion of apoptosis, immune-manipulating mechanisms are now also considered pivotal characteristics of cancer cells [9].

The complex interplay between cancer cells and the host immune response has been deeply investigated over the past few decades, showing that malignant cells have the ability to influence their immune microenvironment, ultimately creating conditions that foster tumour growth and metastasis [10]. Growing evidence suggests that both the innate immune cells (macrophages, neutrophils, dendritic cells, innate lymphoid cells, myeloid-derived suppressor cells and natural killer cells) and the adaptive immune cells (T cells and B cells) contribute to this process when present in the TME [11]. Thus, it is now known that despite exerting a key role in host protection and in cancer cell recognition and eradication, the immune system can also facilitate cancer progression, particularly in advanced stages.

Therefore, new therapies attempting to re-tune and modulate the immune system to target and fight tumours have gained attention. As a result, immunotherapy is now at the forefront of cancer research for the development of novel therapeutics with clinical impact [12,13,14].

BC has previously been considered an immunogenically quiescent or “cold” tumour due to low lymphocyte infiltration, low mutational burden, and limited response rates to anti-programmed cell death 1 (PD-1)/programmed cell death ligand 1 (PD-L1) monotherapy; however, the identification of tumour infiltrating lymphocytes and other immune infiltrates in BC has led to the application of immunotherapies for the disease [15,16,17]. This review summarizes the key findings and emerging approaches in the field of immunotherapy for BC.

## 2. Immunoediting in Breast Cancer

The concept that the immune system could detect and eradicate nascent transformed cells was first suggested by Ehrlich in the early 1900s; however, it did not gain traction until 50 years later, when it was formally introduced as the cancer immunosurveillance hypothesis by Burnet and Thomas [18]. It postulated that lymphocytes were responsible for eliminating continuously arising, nascent transformed cells, leading to the complete regression of the tumour and leaving no clinical hint of its existence. Cancer immunologists had provided insights throughout the 20th century through both clinical observations and experimental data. However, the idea of cancer immune surveillance did not gain widespread acceptance until the 1990s when the development of gene targeting and transgenic mouse technologies and the capacity to produce highly specific blocking monoclonal antibodies (mAb) to particular immune components validated the existence of cancer immune surveillance in both chemically induced and spontaneous tumours [18]. Despite strong evidence supporting the existence of a functional cancer immunosurveillance process, the fact that immunocompetent individuals still developed cancer suggested that a much more complex regulation of the immune environment was involved in cancer progression. Indeed, the cancer immunoediting theory developed by Dunn and colleagues in 2002 refined the original cancer immunosurveillance hypothesis and proposed a dual role of the immune system in cancer progression [11]. The immune system serves not only to protect the host from tumour development, but also to sculpt, or edit, the immunogenicity of tumours that may eventually form. According to this theory, the relation between tumour cells and the immune system is a dynamic process composed of three main phases: elimination, equilibrium, and escape [19].

**Figure 2:**
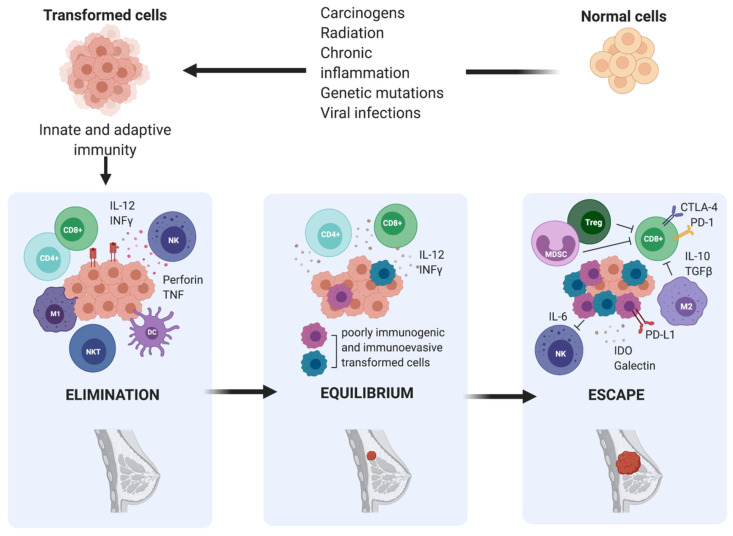
The three phases of cancer immunoediting in breast cancer. Elimination is the first phase of cancer immunoediting. Early in mammary tumorigenesis, acute inflammation induces the activation of innate immunity, including type 1- polarized macrophages (M1), natural killer (NK), and natural killer T cells (NKT), resulting in both tumour cell death and the maturation of dendritic cells (DC), which can prime tumour-specific T cells (CD4^+^ and CD8^+^). Inflammation-related soluble factors, including IL-2, IFNγ, perforin, and TNF, can be found in the TME. This stage is followed by either immune-mediated rejection of incipient tumours or the selection of tumour cell variants, which can induce chronic inflammation. Hence, the persistent cells enter the equilibrium phase. Ultimately, this leads to the escape phase, which results in a complex and immune-tolerant TME, consisting of suppressive immune cells, including regulatory T cells (Treg), type 2- polarized tumour-associated macrophages (M2), and myeloid-derived suppressor cells (MDSC), and inhibitory molecules such as IL-6, IDO, galectin, IL-10, and TGF-β, that allow overt immune escape and tumour progression to occur.

During the elimination phase, immune cells attempt to eliminate cancer cells upon detection. Next, in the equilibrium phase, surviving cancer cells with a resistant or non-immunogenic phenotype are suppressed but not destroyed, resulting in functional dormancy. Subsequently, cancerous cells are in equilibrium with their microenvironment and are more prone to mutate and produce new populations of tumour variants. During the escape phase, the selective pressure of the immune responses promotes the uncontrolled proliferation of sculpted cancer cells with a resistant or a non-immunogenic phenotype, leading to tumour progression and metastasis. According to this theory, the interaction between breast tumours and host immunity is also characterized by immunoediting, which results in a complex and immune-tolerant TME, consisting of suppressive immune cells and inhibitory molecules that allow overt immune escape and tumour progression to occur [20,21] (Figure 2).

## 3. Immune Evasion in Breast Cancer

The escape phase represents the failure of the immune system to eliminate or control transformed cells, allowing surviving tumour cells to grow in an immunologically unrestricted manner. This occurs when genetic and epigenetic changes in the tumour cells confer a selective advantage, giving resistance to immune detection and/or elimination or when the tumour induces a state of immunologic suppression or tolerance in the host, allowing the tumour cells to expand and become clinically detectable [22]. Because both the adaptive and innate compartments of the immune system function in the cancer immunosurveillance network, tumours have to circumvent either one or both arms of immunity in order to achieve progressive growth, evolving a joint strategy of both stealth and camouflage [18].

The mechanisms for tumour escape are varied, but they can be categorized as tumour cell alterations, which directly produce evasion of immune recognition and attack, and the induction of an immunosuppressive microenvironment, achieved through different mechanisms such as the secretion of inhibitory molecules, the recruitment of suppressive immune cells, and the inhibition of immune cells. Combined, these strategies result in a complex and efficient system for immune evasion.

### 3.1. Tumour-Related Immune Evasion

The presentation of tumour-derived antigenic epitopes is the first step allowing the immune system to distinguish between normal and transformed cells and direct the immune response. To facilitate evasion from immune recognition, tumour cells can acquire defects in antigen processing and presentation pathways. This promotes low level of expression of tumour associated antigens at early phases of tumour growth, loss of antigenic epitopes, and low expression or loss of major histocompatibility complex class I (MHC class I) peptide presentation, which permits tumour cell escape from cytotoxic T cells (CTL) [23,24].

A high percentage of human tumours displays downregulation of MHC-I due to epigenetic and/or genetic modifications. Expression levels of human leukocyte antigen (HLA) class I molecules are significantly downregulated in BC due to gene mutation, loss of heterozygosity, and disturbance of transcriptional control [25]. Downregulation of β-2 microglobulin or other components of the pathway, including transporter-associated antigen processing 1 (TAP1) and calnexin, has been found in metastatic brain lesions of BC and is negatively correlated with CTL infiltration [26]. To overcome the attack of CTLs and natural killer (NK) cells, BC cells can also upregulate the non-classical MHC class I molecule, HLA-G, and release soluble natural killer group 2D receptor ligand (NKG2DL) which in turn blocks the activating NKG2D receptor. The results of a tumorigenicity test using an orthotopic xenotransplant BC model indicated that the self-stimulation of natural killer group 2D receptor (NKG2D) could promote BC by increasing angiogenesis and promoting tumour growth, intravasation, and dissemination [27].

Tumour cells that are unable to avoid immune cell detection can develop mechanisms to evade immune-mediated apoptosis, which is induced mainly by the release of cytotoxic granules or the activation of death receptors. The perforin/granzyme and Fas/FasL pathways are the two main effector mechanisms by which CTLs and NK cells mediate antitumor immunity, as they both lead to the activation of the caspase cascade [28]. Any step of the apoptosis pathway can be disturbed, inducing uncontrolled cell proliferation. Regarding the Fas/FasL pathway, the interaction between the death receptors (Fas) and their ligands (FasL), expressed on the activated T lymphocytes, is the first step in the induction of the downstream signal cascade [29]. BC can either increase FasL expression, inducing effector T lymphocytes to die, or downregulate Fas. In particular, the intracellular signalling domain of Fas, the death domain, is frequently deficient in BC [25,30]. Alternatively, BC can evade immune-mediated destruction by upregulating antiapoptotic molecules. Bcl-2 is commonly overexpressed and protects cells against apoptosis by preventing cytochrome c release from mitochondria [31]. Survivin is also increased in BC and is associated with poorer outcome, advanced tumour grade, worse metastasis, and lower survival rate [32]. The decrease in caspase activation is another mechanism used by cancer cells to resist apoptosis. Accordingly, caspase-3 is downregulated in BC [33].

### 3.2. Immunosuppressive Microenvironment

**Figure 3:**
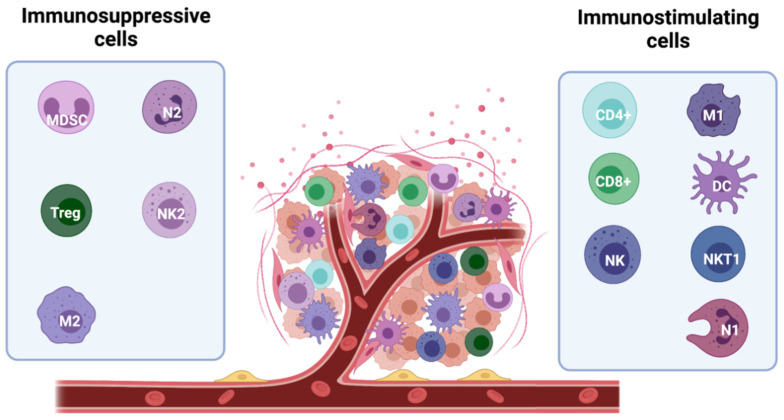
Major players in the immune microenvironment of breast cancer. Subtypes of immune cells can elicit both tumour-promoting and tumour-suppressing effects. The anti-tumour activity is mainly driven by immunostimulating immune cells, including M1 macrophages, CD8^+^ and CD4^+^ lymphocytes, NK and NKT1 cells, and DCs and N1 neutrophils. They secrete cytokines and soluble factors which help fighting the tumour development (including IFNγ, TNFα, IL-1β, IL-2, and IL-12). In contrast, immunosuppressive cells, including myeloid-derived suppressor cells (MDSCs), mast cells (MCs), regulatory T cells (Tregs), type 2- polarized tumour-associated macrophages (M2-TAMs), and N2 tumour-associated neutrophils (N2-TAN) can be recruited to the tumour site counteracting the anti-tumour activity and facilitating tumour growth. These cells release immuno-inhibitory pro-tumour cytokines (TGF-β, VEGF, IL-6, IL-8, and IL-10).

The ability of the tumour mass to expand depends on global factors, such as the induction of an immune-tolerant TME. Infiltration of tumours by immunostimulating immune cells, such as a subset of macrophages (M1-TAMs), lymphocytes, NK cells, innate lymphoid cells (ILCs), dendritic cells (DCs), and eosinophils is crucial for tumour control [17]. However, immunosuppressive cells, including myeloid-derived suppressor cells (MDSCs), mast cells (MCs), regulatory T cells (Tregs), type 2- polarized tumour-associated macrophages (M2-TAMs), and N2 tumour-associated neutrophils (N2-TAN) can be recruited to the tumour site to inhibit the anticancer immune response and create an immune-tolerant TME [34] (Figure 3).

Treg cells may be considered the ‘centre’ of the immunosuppressive network. Under physiological conditions, they play a central role in maintaining immune homeostasis and self-tolerance, dampening inflammation, and preventing autoimmunity. They are characterized by the expression of markers such as CD4, CD25, and forkhead box P3 (FOXP3), which plays an important role in Treg cell development and function. Treg cells recruitment in a range of human cancer types is mediated by several soluble factors, such as C-C motif chemokine ligand 22 (CCL22), C-C motif chemokine ligand 28 (CCL28), and C-X-C motif chemokine ligand 12 (CXCL12), produced by tumour cells, cancer associated fibroblasts and immunosuppressive cells [35]. Interestingly, the expression of CXCL12 and its receptor C-X-C motif chemokine receptor 4 (CXCR4) is increased by hypoxia, which could further promote Treg infiltration in breast tumours, especially in the basal-like subtype [36].

T regs are responsible for suppressing the priming, activation, and cytotoxicity of other effector immune cells, such as T helper 1 (Th1) CD4^+^ T cells, CTLs, macrophages, NK cells, and neutrophils. They exert their immune suppressive function through contact-dependent mechanisms such as the expression of PDL-1, lymphocyte activation gene 3 (LAG-3), CD39/73, cytotoxic T lymphocyte associated antigen 4 (CTLA4), or PD-1, and through contact-independent mechanisms, which involve the sequestration of interleukin 2 (IL-2) and the production of immune-suppressive molecules such as interleukin 10 (IL-10), transforming growth factor β (TGF-β), prostaglandin E2 (PGE2), adenosine, and galectin [37]. Higher numbers of Tregs have been found in the peripheral blood of BC patients compared with healthy controls and it has been reported that their ability to infiltrate tumours increases with tumour stage and correlates with poor prognosis in invasive BCs [38].

Macrophages can present two different polarizations: the pro-inflammatory M1 type (classically activated) and the anti-inflammatory/immunosuppressive M2-type (alternatively activated) [39]. Both M1- and M2-polarized macrophages have been identified in the TME [40]. However, the immunosuppressive phenotype is predominant in the BC TME and it is associated with poor prognostic outcome, decreased relapse-free survival, and overall survival [41,42,43,44]. It has been shown that M2-TAMs promote tumour growth, angiogenesis, invasion, metastasis, and resistance to therapy [42,45]. TAMs release several factors, such as vascular endothelial growth factor (VEGF), platelet-derived growth factor (PDGF), basic fibroblast growth factor (bFGF), as well as many signalling molecules, including epidermal growth factor (EGF), matrix metalloproteinases (MMPs), CCL2, C-C motif chemokine ligand (CCL18), and macrophage colony stimulating factor (M-CSF), which can stimulate angiogenesis and induce epithelial–mesenchymal transition (EMT), invasion, and metastasis [46,47]. Moreover, M2-TAM-derived TGF-β and IL-10 can suppress CD8^+^ T cell functions, reducing their cytotoxic activity [46,48], while the high levels of enzymes such as arginase 1 (ARG1) and indoleamine 2,3-dioxygenase 1 (IDO1) can deplete the TME of the amino acids arginine and tryptophan which are essential for T and NK cells proliferation and survival [47].

Similar to TAMs, neutrophils can show two distinct phenotypes under different stimuli: N1-tumour associated neutrophils (N1-TANs) and N2-tumour associated neutrophils (N2-TANs) with anti-tumour and pro-tumour functions, respectively. Increased levels of blood neutrophils have been described in various tumours including BC and a high neutrophil-to-lymphocyte ratio has been associated with a poor outcome [49]. Similar to M2-TAMs, N2-TANs are able to promote tumour growth, angiogenesis, invasion, and metastasis through the release of several factors. CCL2 and C-C motif chemokine ligand 17 (CCL17) support tumour growth by recruiting CD4^+^ Treg cells and macrophages [50], while VEGF and MMP-9 stimulate angiogenesis and tumour cells migration [51,52,53]. In addition to recruiting immune-suppressive cells, TANs were also reported to inhibit NK cell-mediated clearance of tumour cells [54] and dampen the survival and cytotoxic effect of CD8^+^ T cells [55,56]. Moreover, neutrophils can also assist the formation of a cancer premetastatic niche in distant organs [56].

Another class of immune cells with immunosuppressive activity is MDSCs, a heterogenous population of immature myeloid progenitor cells. Based on the different cell surface antigen expression, two main populations have been described: granulocytic or polymorphonuclear MDSCs (PMN-MDSCs), and monocytic MDSCs (M-MDSCs) [57]. Both M-MDSCs and PMN-MDSCs are recruited to tumour sites by tumour-derived cytokines: CCL2 and C-C motif chemokine ligand 5 CCL5 for M-MDSCs, C-X-C motif chemokine ligand 1 (CXCL1), C-X-C motif chemokine ligand 2 (CXCL2), C-X-C motif chemokine ligand 5 (CXCL5), C-X-C motif chemokine ligand 6 (CXCL6), C-X-C motif chemokine ligand 8 (CXCL8), CXCL12, CCL2, C-C motif chemokine ligand 3 (CCL3), and C-C motif chemokine ligand 15 (CCL15) for PMN-MDSCs [58]. MDSC-mediated immune suppression is due to different mechanisms, which all induce anergy of effector immune cells and promote the recruitment of immunosuppressive cells [8]. They produce ARG1, reactive oxygen species (ROS), reactive nitrogen species (RNS), cyclooxygenase-2 (COX-2), PGE2, IL-10, and TGF-β, which induce severe anergy of effector immune cells, such as NK cells and CD8^+^ T cells [59]. Moreover, secretion of IL-10 and TGF-β is also associated with the differentiation and expansion of Tregs and to M2-like TAM polarization [60]. In addition, MDSCs upregulate the expression of Programmed cell death protein ligand 1 (PD-L1), blocking the anti-tumour T cell-mediated activity via an interaction with the programmed cell death protein 1 (PD-1) receptor of these cells [61]. Several studies have shown that MDSCs are associated with poor prognosis in BC patients. Indeed, MDSCs are more enriched in triple-negative BC (TNBC) patient samples compared to non-TNBC [62], and high levels of circulating MDSCs significantly correlate with liver and bone metastases and higher levels of circulating tumour cells [63].

The immunosuppressive TME is highly sculpted by soluble cytokines and immunomodulators. There is ample evidence that tumour cells, along with immunosuppressive cells, have evolved to produce immunosuppressive factors. Although released at the primary tumour site where they can exert a local effect, these secreted factors also have systemic effects on immune function, as a result of transport to local lymph nodes and peripheral tissues. A variety of tumour-derived soluble factors has been linked to the building of complex immunosuppressive networks, including VEGF, IL-10, TGF-β, prostaglandin E2, soluble ligands (soluble MHC class I polypeptide-related sequence A (MICA), UL16 binding proteins (ULBPs) and decoy receptors, as reminded before, or effector molecules [27]. In particular, TGF-β is a functional bidirectional cytokine and the most studied immunosuppressive cytokine induced by BC. It has antiproliferative activity in the early phases of cancer, acting through cell cycle arrest [64]. However, alterations of TGF-β signalling have been detected in BC, especially in the later stages [65]. Interleukins such as IL-6, -18, -19, -20 and -23, TNFα, and galectin can also provide a favourable microenvironment for tumour growth and foster the proliferation and progression of BC [27].

An additional strategy by which BC cells are able to evade immune destruction is mediated by cell–cell contact. Tumour cells and some immunosuppressive cells can hijack the ‘immune checkpoint’ pathways. Under normal physiological conditions, immune checkpoints are crucial for the maintenance of self-tolerance and to protect tissues from damage when the immune system is responding to pathogenic infection. However, the expression of immune-checkpoint proteins can be dysregulated by tumours as an important immune resistance mechanism [66].

The best characterized immune-checkpoint receptors are CTLA-4 and PD-1, which bind to CD80/CD86 and PD-L1/PD-L2 ligands, respectively, to initiate checkpoint signalling and cytotoxic T cell inhibition [67].

PD-1 is a negative regulatory receptor expressed by activated T and B cells. It binds two ligands, PD-L1 (B7-H1, CD274) and PD-L2 (B7-DC, CD273), which belong to the B7 protein family. In addition, B7-H3 and B7-H4 have been recently identified as PD-1 ligands, being upregulated on tumour cells or tumour-infiltrating cells [68]. PD-1 is highly expressed by tumour infiltrating lymphocytes (TILs) from many cancers, while PD-1 ligands, in particular PD-L1, are commonly upregulated on the tumour cell surface from many different human tumours and on myeloid cells in the tumour microenvironment.

The PD-1/PD-L1/PD-L2 axis can induce anergy and/or apoptosis of PD-1^+^ T cells, attenuating the anti-tumour immune response and promoting Treg immunosuppressive activity [69,70]. A higher PD-L1 expression has been observed in HER2^+^ BC and TNBC subtypes [71,72]. In addition, it has been demonstrated that expression levels of B7-H1 increased in TNBC and the elevated expression level of B7-H4 was correlated with the negative status of hormone receptors and the positive state of HER2 [73,74].

CTLA4 is expressed mainly on T cells where it primarily regulates the amplitude of the early stages of T cell activation. It is immediately upregulated following T-cell receptor (TCR) engagement and dampens TCR signalling outcompeting the costimulatory molecule CD28 (which provide positive costimulatory signals, strongly amplifying TCR signalling to activate T cell) in binding the B7 ligands B7-1 (CD80) and B7-2 (CD86), for which CTLA4 has higher avidity and affinity. Through this mechanism, CTLA4 attenuates the positive co-stimulation by CD28 and thus limits kinase signals that are induced by TCR and CD28 [75].

BC cells can also induce PD-1 expression in other immune cell populations, enhancing their immunosuppressive function. In particular, tumour cells can modulate PD-L1 expression on MDSCs through the release of cytokines such as IFN-γ [76]. Moreover, it has been shown that Tregs also, accumulated in BC microenvironment, express high levels of CTLA-4 and PD-1, sustaining T cell inhibition [77].

Beyond PD-1 and CTLA-4, many other immune checkpoint receptors have been identified. In particular, lymphocyte activating 3 (LAG3), T cell membrane protein 3 (TIM3), and T cell immunoglobulin and ITIM domain (TIGIT) co-inhibitory receptor have been proposed as prognostic markers in BC, together with CD47 [78,79,80]. CD47 is expressed on the surface of several types of cancer cells and functions as an anti-engulfment signal that protects cells from phagocytosis by macrophages [81]. It is highly expressed on TNBC, and it has been associated with EMT and poor prognosis [82].

## 4. Recent Advances in Breast Cancer Immunotherapy

**Figure 4:**
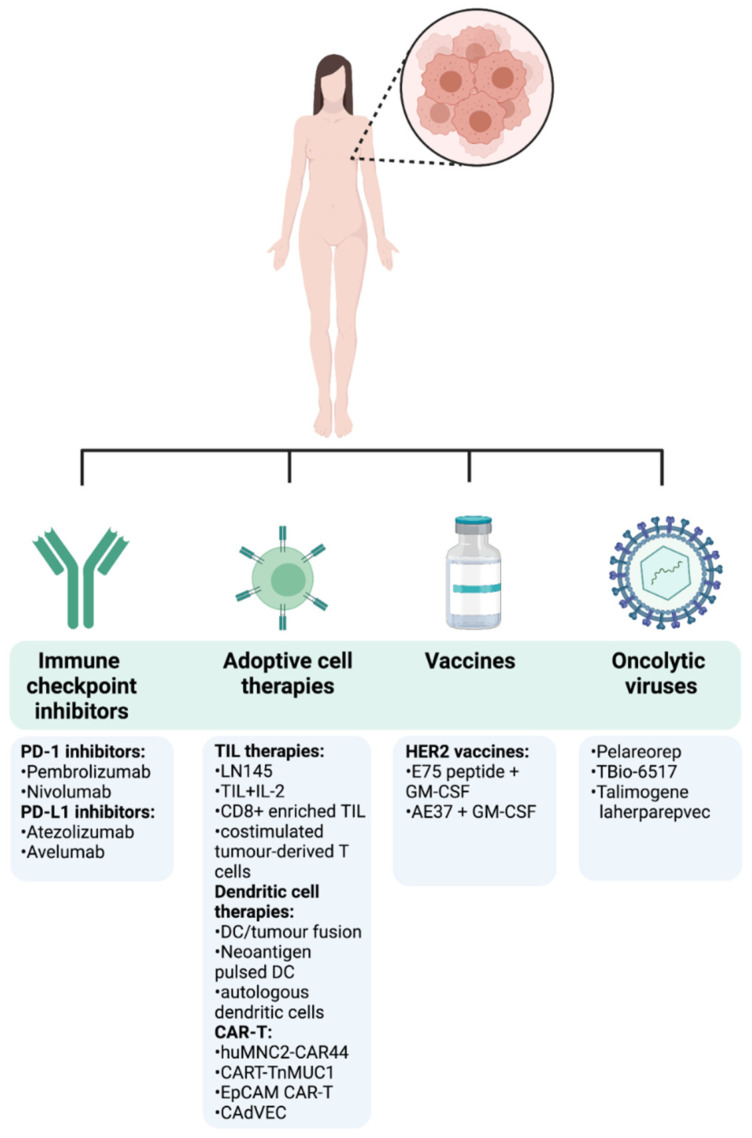
Immunotherapy approaches in breast cancer. The first cancer immunotherapy treatment entered the clinical practice for BC patients in September 1998 with the FDA approval of the humanized HER2 monoclonal antibody trastuzumab for the treatment of metastatic BC patients with HER2 overexpression and/or gene amplification. This represented a milestone in the treatment of BC and has been followed by other different anti-HER2 monoclonal antibodies including lapatinib, neratinib, gefitinib, or afatinib, delivered as monotherapy or in combination with conventional treatments [83]. After that, despite BC immune landscape being dynamic and heterogeneous among tumour stages, subtypes, and disease settings, an emerging body of preclinical and clinical data started to emerge, highlighting the effectiveness of immunotherapies in BC. Following the encouraging long-term success of checkpoint inhibitors in the treatment of different tumours, the FDA approved the first checkpoint inhibitor immunotherapy drug, the anti-PD-L1 antibody atezolizumab in combination with chemotherapy (Abraxane) for the treatment of triple-negative, metastatic BC patients with positive PD-L1 protein expression as a result of the findings from the Phase III double-blind IMpassion130 trial (NCT02425891). However, the limited complete response rates and the immune-mediated serious adverse events encouraged the search of new immunotherapeutic strategies, including adoptive cell transfer and oncolytic viruses, either as monotherapy or in combination with other treatments. A summary of the most recent and relevant immunotherapy approaches being currently under investigation in clinical trials for the treatment of BC is reported.

Thanks to further understanding of the importance of the immune TME and how it modulates cancer progression, immunotherapy is at the forefront of novel cancer therapies. A common theme of current clinical drug trials is their attempt to re-tune and enhance the immune responses that have been lost and suppressed in cancer. Many different strategies can be pursued in order to harness the immune system to target tumours (Figure 4). Below, we provide the most recent clinical evidence supporting the use of several immunotherapy strategies in metastatic as well as early breast cancer (Table 1).

### 4.1. Clinical Efficacy of Immune Checkpoint Inhibitors in Breast Cancer

Immunotherapy, in the form of PD-1 and PD-L1 inhibitors, is now a therapeutic option in BC that can improve survival amongst responders. Most of the clinical efficacy is focused on TNBC which is well-established as being the most immunogenic. The most common BC subtype, ER/PR positive, has demonstrated very limited activity with current immunotherapy approaches. The phase Ib, multicohort, Keynote-028 trial demonstrated an objective response rate (ORR) of only 12% amongst heavily pre-treated ER positive, HER2 negative metastatic breast cancer patients, treated with pembrolizumab monotherapy [84].

More encouraging results came from the early phase trials of immunotherapy in triple negative metastatic breast cancer. The phase Ib, Keynote-012 trial demonstrated an ORR of 18.5% with durable responses amongst patients with triple negative breast cancer, whose tumour was PD-L1 positive (Combined positive score (CPS) ≥ 1%), treated with pembrolizumab monotherapy [85]. However, less encouraging results came from the phase II, Keynote-086 trial, where previously treated metastatic triple negative patients demonstrated an ORR of only 5.3% in the entire cohort population, and only 5.7% amongst the 60% of patients with PD-L1 positive tumours (although in cohort B—1st line TNBC PD-L1^+^ group, ORR = 23.1%) [86]. Furthermore, results from the JAVELIN trial, a phase Ib trial of avelumab monotherapy in multiple solid tumour types, revealed an ORR of 22% in the subgroup of patients with PD-L1 positive TNBC [96]. Despite the conflicting results around the response rate of PD-1 inhibitors in TNBC, the durable responses noted in this trial led to larger scale trials aiming to investigate the efficacy of immunotherapy in triple negative disease. Keynote-119, a phase III randomized trial of pembrolizumab vs. investigator’s choice of single-agent chemotherapy in patients previously treated with one or two previous lines of chemotherapy for metastatic disease, failed to demonstrate any superiority of pembrolizumab vs. standard of care chemotherapy, neither in the intention-to-treat population, nor in the PD-L1 positive subgroups (CPS ≥ 10 and CPS ≥ 1) [88].

Given the limited activity of immunotherapy in TNBC disease as a monotherapy, the efficacy of PD-1/PD-L1 inhibitors was further investigated in combination with chemotherapy. The Keynote-150 phase Ib/II trial, investigating the combination of pembrolizumab and eribulin, demonstrated an ORR of 22% in previously treated patients with a median overall survival (OS) of 15 months [90]. Clinical activity was noted regardless of PD-L1 status. Similarly, in a phase Ib trial of atezolizumab and nab-paclitaxel in previously treated TNBC patients, the ORR was 39% and median OS was 14.7 months [104]. In the TONIC trial, an adaptive, noncomparative phase II trial, 67 patients with metastatic triple-negative breast cancer were randomly assigned to receive nivolumab without induction or with a 2-week low-dose chemotherapy induction, or with irradiation (3 × 8 Gy), all followed by nivolumab. In the overall cohort, the objective response rate (ORR) per response evaluation criteria in solid tumours (iRECIST) was 20%. The majority of responses were observed in the cisplatin (ORR = 23%) and doxorubicin (ORR = 35%) cohorts. Translational research from the TONIC trial revealed a proof-of-concept upregulation of immune-related genes involved in PD-1/PD-L1 and T-cell mediated cytotoxic pathways after an immune-induction strategy [91].

Most of the clinical data above demonstrate a relatively limited activity of immunotherapy as a monotherapy and in combination with chemotherapy, beyond the first line setting in metastatic TNBC. Therefore, the large, randomized phase III trials which eventually established the efficacy of immunotherapy in combination with chemotherapy in TNBC disease focused on patients previously untreated for metastatic disease. The IMpassion-130 trial was a phase III randomized trial of atezolizumab and nab-paclitaxel vs. placebo and nab-paclitaxel in previously untreated metastatic TNBC patients. The benefit was most pronounced amongst the PD-L1 positive population (40% of enrolled patients) with an OS of 25.0 months vs. 15.5 months for nab-paclitaxel [95]. In contrast to the Impassion-130, a similarly designed phase III trial, IMpassion-131 demonstrated no survival benefit with the addition of atezolizumab to weekly paclitaxel, neither in the intention-to-treat, nor in the PD-L1 positive population [94]. Although this was a concern in terms of the conflicting results seen, one of the reasons postulated for the absence of benefit in the IMpassion-131 was the regular use of steroids as pre-medication with paclitaxel. The IMpassion-130 was eventually the basis for the relevant FDA and EMA approvals of atezolizumab-nab-paclitaxel as 1st line treatment in PD-L1 positive metastatic TNBC. Furthermore, Keynote-355, another practice-changing phase III randomized trial enriched in high PD-L1 patients, revealed a significant PFS benefit of 9.7 vs. 5.6 months for the addition of pembrolizumab to standard of care chemotherapy in first line metastatic TNBC PD-L1^+^ (CPS ≥ 10) patients [92]. The above data led to a similar FDA approval for pembrolizumab and chemotherapy in previously untreated metastatic TNBC patients with PD-L1^+^ tumours.

The efficacy of PD-1/PD-L1 inhibitors has also been studied in the early, neoadjuvant setting in TNBC. The phase II, I-SPY2 trial, was an adaptively randomized phase II platform trial for high-risk, stage II/III BC. The addition of pembrolizumab to standard-of-care neoadjuvant chemotherapy resulted in higher pathological complete response rates (pCR) in HER2 negative and ER positive/HER2 negative sub-groups; however, the most prominent benefit was seen in the TNBC subgroup with a pCR rate of 60% for pembrolizumab vs. 22% for the control [93]. Keynote-522 was a phase III randomized study investigating the addition of pembrolizumab to standard of care neoadjuvant chemotherapy (carboplatin-paclitaxel followed by doxorubicin/epirubicin-cyclophosphamide) in stage II/III early TNBC. In this trial, there was a significant increase in the pathological complete response rate from 51% in the placebo group to 65% in the pembrolizumab group, while the preliminary analysis also revealed a benefit for event-free survival [89]. On the basis of Keynote-522, the FDA approved pembrolizumab in the neoadjuvant setting in early stage TNBC. A somewhat smaller randomized study, the IMpassion-031, examined the impact of the addition of atezolizumab to standard neoadjuvant chemotherapy in early TNBC compared to placebo. Similarly to Keynote-522, the pCR rate increased from 41% (placebo) to 58% with atezolizumab. Interestingly, patients with positive PD-L1 expression demonstrated a higher pCR rate with atezolizumab (69%), as opposed to 49% with placebo [97].

The data above highlight the improved efficacy of the addition of PD-1/PD-L1 inhibitors to standard of care chemotherapy, either in the early or metastatic setting. Most of the benefit appears to be limited to high PD-L1 expressing tumours in both indications.

### 4.2. Adoptive Cell Therapy Role in Breast Cancer

Adoptive cell therapy is being investigated in early phase clinical trials in breast cancer; however, there is currently no conclusive, convincing evidence of efficacy as of yet. TIL therapy is one particular strategy of adoptive cell therapy where tumour sections are cultured in the presence of IL-2 in order to expand T cells. Tumour cells are analysed through whole exome sequencing or transcriptome analysis to identify tumour neoantigens specific to the tumour of an individual patient. Immature antigen-presenting cells are then transfected with genes expressing the specific tumour neoantigens. TILs are then co-cultured with dendritic cells, specifically expressing the pre-selected tumour neoantigens, and are subsequently selected on the basis of a positive immune response that they provoke. TILs are then infused to the patient, often with IL-2 treatment to promote a robust immune response [105]. Zacharakis et al. recently presented a case of a chemorefractory hormone receptor (HR)-positive metastatic breast cancer patient who was treated with tumour-infiltrating lymphocytes (TILs) reactive against mutant versions of four proteins, SLC3A2, KIAA0368, CADPS2, and CTSB. Adoptive transfer of these mutant-protein-specific TILs in conjunction with IL-2 and checkpoint blockade mediated a complete durable regression of metastatic breast cancer in this case [106]. There are currently several early phase trials investigating the efficacy of TIL therapy in TNBC, HER2 positive, and luminal-type metastatic breast cancer (NCT04111510, NCT01462903, NCT01174121, NCT00301730).

Dendritic cell administration is another strategy which is currently being investigated in many tumour types, including metastatic breast cancer. The capability of dendritic cells to stimulate a T-cell cytotoxic response and T-cell memory render them particularly appealing as a mediator of immune response against tumour neo-antigens. Immature peripheral blood stem cells are effectively differentiated towards a dendritic-phenotype. They are then pulsed with tumour cell lysates containing multiple neoantigens in the presence of stimulatory cytokines. The dendritic cells then mature into fully differentiated, specific-to-tumour-neoantigen cells before they are subsequently infused back to the patient [105]. Only a small number of studies have been conducted where they demonstrate a very limited effect of dendritic cell therapy in breast cancer. A phase I trial, where a dendritic vaccine was developed via the fusion of patients’ dendritic cells with co-cultured tumour cells, demonstrated two cases of disease regression in patients with metastatic breast cancer, including a patient with a near complete response [98]. There are currently several ongoing early phase clinical trials investigating the efficacy of dendritic cell therapy in all subgroups of breast cancer, both in the perioperative and metastatic setting (NCT04105582, NCT03630809, NCT03450044, NCT04348747).

Chimeric antigen receptor-T-cell therapy (CAR-T) is another form of adoptive cell therapy which has revolutionised the management of diffuse large B-cell lymphoma, indolent lymphomas, and acute lymphoblastic leukaemia with surprisingly durable responses in heavily pre-treated patients with otherwise minimal remaining treatment options, and has therefore become standard of care in the management of the above diseases [107,108,109]. CAR-T cell therapy is based on harvesting T-cell from patients’ peripheral blood, and genetically modifying them to express a chimeric antigen receptor against a tumour antigen which is widely and specifically expressed on the surface of cancer cells. The cells are then expanded in vitro and reinfused to the patient following treatment with a lymphodepletion regimen consisting of fludarabine and cyclophosphamide. CAR-T cells are capable of specifically recognizing and attaching to the tumour neoantigen via the genetically modified receptor, eliciting a robust cytotoxic immune response through the transmembrane activation of downstream signalling pathways, leading to the stimulation of their cytotoxic activity [110]. One of the tumour neoantigens that is currently extensively investigated in early phase CAR-T clinical trials (NCT04020575 and NCT04025216) is the Mucin-1 glycoprotein (MUC1) which is abundantly expressed in the surface of TNBC cells, whereas no significant expression is noted in normal breast tissue [111]. Epithelial cell adhesion molecule (EpCAM) is another transmembrane glycoprotein that is overexpressed in various cancers including breast [112]. The NCT02915445 trial is currently recruiting patients with advanced breast cancer, investigating the efficacy and safety of anti-EpCAM CAR-T cell therapy in this setting. An oncolytic Adenovirus in combination with HER2-specific autologous CAR-T cell therapy is recruiting HER2 positive metastatic breast cancer patients as part of the phase I NCT03740256 trial. We are currently nowhere near having conclusive results regarding the safety or efficacy of CAR-T cell therapy in breast cancer; however, recent results from a phase I clinical trial which investigated the safety of CAR-T cells directed against c-met (anti-c-met-CAR-T cells), intratumorally injected into patients with TNBC, demonstrated that c-Met-CAR-T cell therapy was well-tolerated by patients and elicited an inflammatory response within TNBC tumours. It had no evidence of drug-related adverse effects greater than grade 1, thus providing encouraging proof-of-concept evidence [113]. There are several other therapeutically appealing tumour neoantigens that have demonstrated relevant specificity for breast cancer cells based on pre-clinical evidence, and for which a CAR-T cell strategy may be feasible in the future, including chondroitin sulfate proteoglycan 4, receptor tyrosine kinase EGFR, folate receptor alpha, disialoganglioside GD2, intracellular adhesion molecule-1, integrin α_v_β_3_, mesothelin, c-MET, natural killer group 2, member D, receptor tyrosine kinase–like orphan receptor 1,stage-specific embryonic antigen-4, tumour endothelial marker 8, trophoblast cell-surface antigen 2, and others [110].

### 4.3. Cancer Vaccines in Breast Cancer

Cancer vaccines are generally based on the administration of cancer-related antigen peptides to patients, aiming to provoke an immune response or induce immune surveillance to prevent a future recurrence of the disease. Few clinical data exist around the use of cancer vaccines in the management of breast cancer. Given the importance of targeting the HER2 receptor in HER2 positive disease, a small number of cancer vaccine trials focusing on HER2 as the basis for a cancer vaccine have been conducted. A large, randomized, placebo-controlled phase III study investigating the efficacy of a HER2-related peptide, nelipepimut-S (NP-S) plus GM-CSF vaccine, recruited patients with node positive, HER2 low expressing tumours in the adjuvant setting, and although well tolerated, it resulted in no difference in disease-free survival [101]. A phase II randomized study investigating the same peptide in the adjuvant setting again, recruited patients with HER2 low expressing tumours and TNBC tumours. Patients received trastuzumab for 1 year and were randomized to placebo (GM-CSF, control) or nelipepimut-S (NPS) with GM-CSF. There was no difference in DFS for the HER2 low expressing subgroup, whereas there was an apparent benefit in DFS for the TNBC patients [102]. In a different randomized trial in the adjuvant setting, AE37 and GP2, two other HER2 derived peptide vaccines were investigated in a four-arm, prospective, randomized, single-blinded, multi-center phase II trial; the AE37 arm showed no difference in DFS overall; however, subgroup analyses showed a trend towards benefit in advanced stage (*p* = 0.132, HR 0.573 CI 0.275–1.193), HER2 under-expression (*p* = 0.181, HR 0.756 CI 0.499–1.145), and triple-negative breast cancer (*p* = 0.266, HR 0.443 CI 0.114–1.717). The GP2 arm had no significant difference in DFS as compared to the control arm, but on subgroup analysis, HER2 positive patients had no recurrences with a trend toward improved DFS (*p* = 0.052). In conclusion, no definitive results are currently available around cancer vaccination as a strategy in the management of breast cancer disease, and given the controversy of results so far, further large scale clinical trials are required to draw conclusive results.

### 4.4. Oncolytic Viruses in Breast Cancer

There is an extensive amount of pre-clinical evidence supporting the efficacy of the strategy of oncolytic viruses in various tumour types; however, there have been very few examples of this approach successfully entering clinical practice. One example would be Talimogene laherparepvec for the local treatment of unresectable cutaneous, subcutaneous, and nodal lesions in patients with melanoma recurrent after initial surgery [114,115]. Oncolytic virus-mediated oncolysis causes the release of damage-associated molecular patterns (DAMPs) and pathogen-associated molecular patterns (PAMPs) together with a natural repertoire of tumour-specific (TSA) or tumour-associated (TAA) antigens. Together, DAMPs/PAMPs and TAA/TSA provide the key signals to dendritic cells to initiate tumour-specific adaptive immune response [115]. A randomized phase II study of weekly paclitaxel with or without pelareorep, a serotype 3 reovirus, in patients with metastatic breast cancer, did not show a difference in PFS (the primary endpoint) or ORR. However, there was a significantly longer overall survival for the combination [103]. There is an active Phase II trial currently recruiting breast cancer patients to extend this study, by combining Pelareorep and paclitaxel with the anti-PD-L1 antibody, Avelumab (NCT04215146). In conclusion, oncolytic virus strategies are in very early phase development with several actively recruiting studies, some of which in combination with PD-1/PD-L1 inhibitors (NCT04102618, NCT04301011, NCT04185311).

## 5. Conclusions

Sixty years ago, the immune system was thrust into the cancer spotlight for the first time. It was rightly suggested that it could protect against cancer, but we now know that the relationship between cancer and immunity is much more complex and multifaceted, and that the tumour microenvironment is the interface between the cancer and immune cells interaction. Alongside being appointed as the ‘Breakthrough of the Year’ in 2013, cancer immunotherapies have accumulated many promising results over the last 50 years showing that despite heavy immunoediting, the immune system in cancer is not beyond the modulation of novel therapeutics. Reconstituting the immune system to eliminate cancer is a pathway that researchers have explored for the treatment of a wide range of malignancies. After the FDA approval of trastuzumab for the treatment of metastatic BC patients with HER2 overexpression and/or gene amplification, an emerging body of preclinical and clinical data has started to come up, highlighting the effectiveness of immunotherapies in BC. In this review, we have highlighted only a few of the preclinical approaches and ongoing clinical trials in breast immuno-oncology. Currently, there are hundreds of ongoing clinical trials in BC with many of these trials combining immuno-oncology agents and/or standard of care regimens. Despite the huge advances made in this field, there is still a strong need for further research to identify biomarkers useful to select patients suitable for immunotherapies and predict their response to the treatments. Understanding the complex relationship between cancer and the immune microenvironment and unravelling the role of the different components of the innate and adaptive immune system will certainly help to develop more effective immunotherapy strategies against BC.

## Figures and Tables

**Table 1:** Summary of immunotherapy clinical trials in breast cancer.

**Therapy**	**Trial Identifier**	**Phase**	**Intervention**	**Breast Cancer** **Subtype**	**Start Date** **(Estimated Completion Date)**	**Ref**
**Immune checkpoint inhibitors**						
**PD-1**	NCT02054806 (Keynote-028)	Ib	Pembrolizumab	ER+/HER2-PD-L1+ aBC	17 February 2014 (30 April 2021)	[84]
	NCT01848834 (Keynote-012)	Ib	Pembrolizumab	PD-L1 + mTNBC	7 May 2013 (30 June 2020)	[85]
	NCT02447003 (Keynote-086) Cohort A	II	Pembrolizumab	mTNBC ≥ 1 systemic therapy	11 June 2015 (31 January 2020)	[86]
	NCT02447003 (Keynote-086) Cohort B	II	Pembrolizumab	mTNBC PD-L1 + 1st line	11 June 2015 (31 January 2020)	[87]
	NCT02555657 (Keynote-119)	III	Pembrolizumab vs. chemotherapy	mTNBC	13 October 2015 (10 November 2020)	[88]
	NCT03036488 (Keynote-522)	III	Pembrolizumab + Chemotherapy vs. Placebo + Chemotherapy	Stage II/III TNBC 1st line	7 March 2017 (30 September 2025)	[89]
	NCT02513472 (Keynote-150)	Ib/II	Eribulin Mesylate + Pembrolizumab	mTNBC ≤ 2nd line	28 August 2015 (9 April 2021)	[90]
	NCT02499367 (TONIC)	II	Nivolumab Immune induction vs. no induction	mTNBC < 3 lines of therapy	August 2015 (August 2022)	[91]
	NCT02819518 (Keynote-355)	III	Pembrolizumab + chemotherapy vs. placebo + chemotherapy	Locally recurrent inoperable TNBC/mTNBC 1st line	27 July 2016 (12 January 2022)	[92]
	NCT01042379 (I-SPY2)	II	Pembrolizumab + chemotherapy vs. placebo + chemotherapy	High-risk, stage II/III BC	1 March 2010 (December 2031)	[93]
**PD-L1**	NCT03125902 (IMpassion-131)	III	Atezolizumab + paclitaxel vs. placebo + paclitaxel	Locally advanced inoperable TNBC/mTNBC 1st line	25 August 2017 (2 December 2021)	[94]
	NCT02425891 (IMpassion-130)	III	Atezolizumab + nab-paclitaxel vs. placebo + nab-paclitaxel	Locally advanced/mTNBC 1st line	23 June 2015 (30 August 2021)	[95]
	NCT01772004 (JAVELIN)	Ib	Avelumab	mBC	31 January 2013 (16 December 2019)	[96]
	NCT03197935 (IMpassion-031)	III	Atezolizumab + chemotherapy vs. placebo + chemotherapy	Stage II-III TNBC	24 July 2017 (21 October 2022)	[97]
**Adoptive cell therapies**						
**TIL** **Therapy**	NCT04111510	II	LN-145	mTNBC 1–3 lines of therapy	23 December 2019 (January 2022)	n/a
	NCT01462903	I	Tumour infiltrating lymphocytes + IL-2	Breast Carcinoma	September 2011 (December 2014)	n/a
	NCT01174121	II	CD8+ Enriched TIL vs. unselected TIL vs. unselected TIL + pembrolizumab	Metastatic BC ≥ 2 lines of therapy	26 August 2010 (27 December 2024)	n/a
	NCT00301730	I	Costimulated tumour-derived T cells	mBC	October 2005 (n/a)	n/a
**Dendritic cell** **Therapy**	n/a	I	DC/tumour fusion	mBC	July 1999 (March 2002)	[98]
	NCT04105582	I	Neo-antigen pulsed DC	BC	1 August 2019 (1 March 2022)	n/a
	NCT03630809	II	HER2 DC1 Vaccine	HER2+	10 January 2019 (December 2024)	n/a
	NCT03450044	I/II	Autologous dendritic cells + chemotherapy	IDC TNM IIA-IV	January 2014 (August 2018)	[99]
	NCT04348747	IIa	anti-HER2/3 dendritic cell vaccine + Celecoxib + Pembrolizumab + IFN alpha-2b + Rintatolimod	Brain metastases from TNBC or HER2 + BC	1 October 2021 (1 October 2024)	n/a
**CAR-T**	NCT04020575	I	huMNC2-CAR44 CAR T cells	Metastatic HR+ (≥3 lines), HER2+ (≥3 lines), TNBC (≥2 lines)	15 January 2020 (15 January 2035)	n/a
	NCT04025216	I	CART-TnMUC1	mTNBC	10 October 2019 (31 October 2036)	n/a
	NCT02915445	I	CAR-T cells recognizing EpCAM	EpCAM + BC	July 2016 (July 2022)	n/a
	NCT03740256	I	CAdVEC	HER2 + BC	14 December 2020 (30 December 2038)	[100]
**Cancer vaccines**						
**HER2** **Vaccine**	NCT01479244 (PRESENT)	III	E75 peptide + GM-CSF or placebo + GM-CSF	T1-T3 HER2 IHC 1+/2 + node + BC	November 2011 (21 September 2016)	[101]
	NCT01570036	II	E75 peptide (KIFGSLAFL) vaccine + GM-CSF vs. placebo + GM-CSF	Disease-free after HER2 1+/2 + BC	21 May 2013 (28 September 2018)	[102]
	NCT00524277	II	AE37 + GM-CSF vs. GP2 + GM-CSF vs. placebo + GM-CSF	Disease-free after Lymph node+ or high-risk lymph node-HER2 + BC	January 2007 (31 March 2017)	n/a
**Oncolytic viruses**						
**Oncolytic** **virus**	NCT01656538	II	Pelareorep + paclitaxel vs. paclitaxel	Advanced BC/mBC	30 July 2012 (14 February 2018)	[103]
	NCT04215146 (BRACELET-1)	II	Pelareorep + paclitaxel + avelumab vs. pelareorep + paclitaxel vs. paclitaxel	HR+/HER2-endocrine refractory mBC	10 June 2020 (January 2024)	n/a
	NCT04102618 (AWARE-1)	Early I	Pelareorep + letrozole vs. pelareorep + letrozole + atezolizumab vs. pelareorep + atezolizumab vs. pelareorep + atezolizumab + trastuzumab	HR+/HER2-, TNBC, HER2+/HR+, HER2+/HR-	29 March 2019 (December 2020)	n/a
	NCT04301011 (RAPTOR)	I/IIa	TBio-6517 vs. TBio-6517 + Pembrolizumab	Locally advanced/metastatic BC	2 June 2020 (30 December 2022)	n/a
	NCT04185311	I	talimogene laherparepvec, nivolumab, ipilimumab	Localized TN or ER+ HER2-BC	10 July 2019 (1 July 2023)	n/a

aBC, advanced breast cancer; BC, breast cancer; CAR, chimeric antigen receptor; EpCAM, epithelial cell adhesion molecule; ER, estrogen receptor; GM-CSF, granulocyte-macrophage colony-stimulating factor; DC, dendritic cell; HER2, human epidermal growth factor receptor 2; HR, hormone receptor; IDC, invasive ductal carcinoma; IFN, interferon; mBC, metastatic breast cancer; mTNBC, metastatic triple-negative breast cancer; PD-1, programmed cell death protein 1; PD-L1, programmed death-ligand 1; TIL, tumour infiltrating lymphocyte; TN, tumour node; TNM, tumour node metastases.
